# Strawberry Plant as a Biomonitor of Trace Metal Air Pollution—A Citizen Science Approach in an Urban-Industrial Area near Lisbon, Portugal

**DOI:** 10.3390/plants13243587

**Published:** 2024-12-23

**Authors:** Carla A. Gamelas, Nuno Canha, Ana R. Justino, Alexandra Nunes, Sandra Nunes, Isabel Dionísio, Zsofia Kertesz, Susana Marta Almeida

**Affiliations:** 1 Centro de Ciências e Tecnologias Nucleares, Instituto Superior Técnico, Universidade de Lisboa, Estrada Nacional 10, Km 139.7, 2695-066 Bobadela, Portugalsmarta@ctn.tecnico.ulisboa.pt (S.M.A.); 2 Instituto Politécnico de Setúbal, Escola Superior de Tecnologia de Setúbal, Campus do IPS, Estefanilha, 2914-508 Setúbal, Portugal; 3 HyLab—Green Hydrogen Collaborative Laboratory, Estrada Nacional 120-1, Central Termoelétrica, 7520-089 Sines, Portugal; 4 Instituto Politécnico de Setúbal, Escola Superior de Ciências Empresariais, Campus do IPS, Estefanilha, 2914-508 Setúbal, Portugal; sandra.nunes@esce.ips.pt; 5 Center for Mathematics and Applications, NOVAMATH, Universidade Nova de Lisboa, 2829-516 Caparica, Portugal; 6 HUN-REN Institute for Nuclear Research (ATOMKI), 4026 Debrecen, Hungary; zsofi@atomki.hu

**Keywords:** strawberry leaves, air biomonitoring, particulate matter, metal accumulation, citizen science

## Abstract

A biomonitoring study of air pollution was developed in an urban-industrial area (Seixal, Portugal) using leaves of strawberry plants (*Fragaria × ananassa* Duchesne ex Rozier) as biomonitors to identify the main sources and hotspots of air pollution in the study area. The distribution of exposed strawberry plants in the area was based on a citizen science approach, where residents were invited to have the plants exposed outside their homes. Samples were collected from a total of 49 different locations, and their chemical composition was analyzed for 22 chemical elements using X-ray Fluorescence spectrometry. Source apportionment tools, such as enrichment factors and principal component analysis (PCA), were used to identify three different sources, one geogenic and two anthropogenic (steel industry and traffic), besides plant major nutrients. The spatial distribution of elemental concentrations allowed the identification of the main pollution hotspots in the study area. The reliability of using strawberry leaves as biomonitors of air pollution was evaluated by comparing them with the performance of transplanted lichens by regression analysis, and a significant relation was found for Fe, Pb, Ti, and Zn, although with a different accumulation degree for the two biomonitors. Furthermore, by applying PCA to the lichen results, the same pollution sources were identified.

## 1. Introduction

Trace metals in atmospheric particulate matter (PM) are of particular interest given their effect on human health [1]. However, only a small number of air monitoring stations measure metal concentrations, and with a poor spatial coverage. In this context, biomonitoring offers advantages, since biomonitors can be spatially distributed and have a low cost [2].

The most commonly used biomonitors are lichens [3], but plant leaves have also been widely used, because they are efficient collectors of atmospheric particles [4] and due to their widespread presence in terrestrial ecosystems [5,6,7,8,9,10,11,12].

Spatial distribution of the accumulation of airborne metals has been mapped through the chemical content of plant leaves across a specific region. Several studies have shown that plant leaves exposed near metallurgical industries can have high concentrations of heavy metals, compared to a background site [12,13,14,15].

Although the vast majority of the studies use plants as passive biomonitors, active biomonitoring methods offer some advantages, as they guarantee a similar exposure period and allow spatially ordered sampling [16]. In particular, active biomonitoring with potted plants is very suitable, as the influence of confounding variables, such as soil, can be diminished.

Moreover, active biomonitoring is particularly suitable to be used in citizen science, which involves citizens and professionals in the same project, “aiming to create a collective action that favors behavioral changes toward the environment” [17,18] and supports complex sustainability transitions [19].

Strawberry leaves have already been used as active PM receptors [4,20], in the framework of two citizen science projects, one implemented in East Flanders (Belgium) [21], and the other in four European cities [22], where strawberry plants were exposed in urban environments, namely on roadsides. Strawberry leaves contain trichomes and have a rugged micromorphology that is important for PM capture [4]. The Saturated Isothermal Remanent Magnetization (SIRM) signal was used on strawberry leaves to monitor ambient [4,20] and indoor [23] PM.

In recent years, there have been complaints from the population of Seixal (Portugal), an urban-industrial area with the influence of steelworks, regarding episodes of dust deposition on properties. Due to these pollution events, the concerns of the citizens have increased regarding the local air quality and its impact on their health [24]. The local council promoted a set of actions to understand the sources contributing to local PM levels, namely the chemical characterization of the settled dust [25], a citizen science air biomonitoring study using lichens [26] and strawberry plants as biomonitors, and the chemical characterization and source apportionment of PM_2.5_ [27]. Common conclusions were drawn in these three previous publications, namely the influence of traffic and the local steel industries.

This work presents the results of the aforementioned citizen science biomonitoring study regarding the use of the strawberry plant (*Fragaria × ananassa* Duchesne ex Rozier) as a biomonitor, since the results relating to the simultaneously exposed lichens were previously published [26]. The present work aimed to do the following: (1) assess if the strawberry plant biomonitor could confirm the main sources of air pollution previously identified in the study area, namely traffic and steelworks [25,26], and to identify the hotspots spatial distribution; (2) assess the reliability of using the strawberry plant as a biomonitor, and to identify the scope of its use in terms of the monitored elements. To achieve these goals, the chemical characterization regarding the content of metals, metalloids, and non-metals of the leaves from the exposed plants at the outdoor of the citizens’ dwellings was conducted by X-ray Fluorescence spectrometry. Source apportionment techniques were then used to identify the main pollution sources. To achieve goal (2), the biomonitoring responses of strawberry leaves and transplanted lichens simultaneously exposed in the study area were compared. Comparisons between lichens and vascular plants as biomonitors of air pollution are scarce in the literature [28], and can point to different accumulation capabilities depending on the metal and the species. This is thus a novel insight brought by this study, considering that the number of studies which used strawberry leaves as biomonitors is very low, and the knowledge on the capabilities and limitations of this biomonitor is lacking in the literature.

The citizen science approach with the strawberry plant biomonitor allowed us to engage the population in the study and reach the societal goal of raising awareness on air quality, while providing reliable information on trace metal contamination.

## 2. Materials and Methods

### 2.1. Study Area

**Figure 1:**
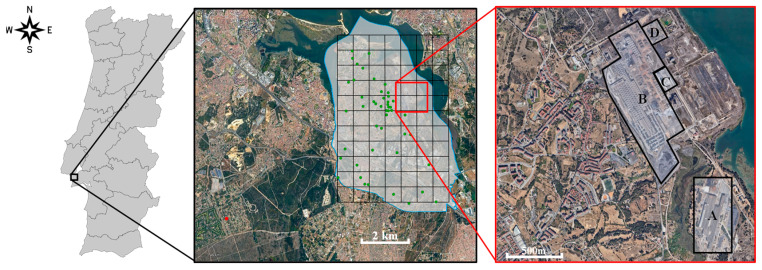
Location of the study area: (**left**) at a national level; (**middle**) spatial distribution of the strawberry plants that could be retrieved after the exposure period (green dots) and the reference background site (red dot); (**right**) location of industries A, B, C, and D.

This study was developed in Aldeia de Paio Pires, municipality of Seixal (Portugal), located in the Lisbon Metropolitan Area (Figure 1). Seixal has a high population density (1744 inhabitants per km^2^ [29]). The study area is crossed by high-density traffic highways and comprises many industries, namely (Figure 1) a steelworks that manufactures galvanized sheet metal with Cr passivation (A), an Electric Arc Furnace steelworks (B), a lime factory (C), and a metal waste treatment factory (D) [26].

### 2.2. Selected Biomonitors

The leaves of strawberry plants (*Fragaria × ananassa* Duchesne ex Rozier, a hybrid that resulted from *F. chiloensis* and *F. virginiana*) were selected as biomonitors of air pollution. Therefore, a set of potted strawberry plants (with same age and uniform potting medium) was acquired from a local supplier.

Additionally, samples of the lichen *Flavoparmelia caperata* (L.) Hale were collected from olive trees at Montargil, a known clean area from inland Portugal. A transplanted lichen was exposed simultaneously next to each potted strawberry plant. Details about the applied methodology and results of the transplanted lichens have been described elsewhere [26].

### 2.3. Exposure Strategy of Biomonitors—Citizen Science Approach

On 1 February 2020, a public event was promoted by the local council in Aldeia de Paio Pires to involve the local population in the biomonitoring study and to provide all the necessary information: (i) how to expose the biomonitors in the outdoor of the dwellings (either on its ground or first floor, at 1.5 m height); (ii) to water the strawberry plants avoiding contact with the leaves; (iii) not to add any fertilizers to the soil or transplant the strawberry plants; (iv) at the end of the exposure period, five sets of three full-grown and undamaged strawberry leaves should be collected by the participants (by touching only the petiole) and sent to the laboratory in a paper envelope.

A total of 78 strawberry plants (Fragaria *× ananassa* Duchesne ex Rozier) and 78 lichen (*Flavoparmelia caperata* (L.) Hale) samples were distributed among the volunteers. These were positioned in a georeferenced grid of 4.55 km × 6.82 km, with 77 cells of 650 m × 620 m, between the coordinates 38°38′53″ N, 9°06′21″ W, and 38°35′9.83″ N, 9°3′12″ W (upper left and lower right corner of the grid), as shown in Figure 1. At each site, one plant and one lichen sample were exposed. Controls were placed outside the grid, in an assumed background site, Verdizela (red dot in Figure 1), characterized by forest and a weekend residential area, with sporadic traffic, at 9 km distance from the industrial area. Considering that citizens that lived nearby the industrial area have already shown concern regarding air quality problems [24], it was expected that those volunteers would show higher engagement, which was confirmed. This allowed us to have a higher sampling density around the steelworks (Figure 1).

The biomonitors were exposed from 1 February to 17 June 2020 (138 days), a period that included the state of emergency decreed in Portugal on 18 March until 4 May due to the COVID-19 pandemic, which included a national lockdown. After the exposure period, leaves from 49 strawberry plants were obtained (due to the withering of some plants), which is above the minimum of 30 suggested for contamination monitoring with leaves [30] and represents a rate of 62.8% in the retrieval of exposed plants.

### 2.4. Chemical Analysis

At the laboratory, the leaves were quickly rinsed (5 s) with demineralized water, freeze-dried, and ground into powder in a ball mill with PTFE capsules, under liquid nitrogen. Pellets with an average thickness of 12 mm were prepared for element characterization by X-ray Fluorescence spectrometry (XRF), to assess the mass fractions of 22 elements (Al, As, Ba, Br, Ca, Cl, Cr, Cu, Fe, K, Mg, Mn, P, Pb, Rb, S, Se, Si, Sr, Ti, Zn, and Zr). XRF analysis was conducted in ATOMKI, Debrecen, Hungary.

A Bruker M4 Tornado type micro-XRF device (high-brilliance X-ray tube with polycapillary X-ray optics; tube parameters: Rh target, 50 kV, 30 W; spot size: ~25 μm for Mo Kα (17.5 keV); 8 excitation filters) was applied to conduct the XRF analysis. For the excitation, the Rh tube was set to 50 kV voltage with a 400 mA current, and no filter was used. A Be windowed XFlash SDD X-ray detector with a 30 mm^2^ surface area recorded the X-ray spectra of the samples. The measurements were performed in vacuum (20 mbar). To obtain the best estimates of the average composition of the pellets, spectra and elemental maps were collected from the largest possible surface area of the sample (~6–8 mm × 6–8 mm). Elemental maps helped to filter out samples with large inhomogeneities. Such pellets were excluded from the analysis. In the case of 10 samples, multi-point analysis was also carried out at 60 randomly selected points per sample. Average concentrations obtained from the point measurements were compared to the results of the area scans. In all cases, concentrations obtained in the two ways were the same within 2 sigma. At least 1 of the 2 or 3 replicate pellets was measured, for each sample. Eight randomly selected samples were re-measured 2 more times on different days, and in the case of 5 pellets, both sides were measured. For 6 samples, all 2–3 replicates were analyzed and compared. In all cases, the obtained concentration data were within uncertainty.

The fundamental parameter (FP) method [31] was utilized for the standardless quantification using the built-in software M-Quant of M4 Tornado version 1.6.614.0 [32]. Cellulose (C_6_H_10_O_5_) was set as the matrix material.

Two plant samples from proficiency tests PTNATIAEA/19 and PTNATIAEA/20 conducted by the International Atomic Energy Agency (http://www.pt-nsil.com/) served as reference materials for the QA/QC of the XRF measurements. Results of XRF analysis of the reference samples and instrumental detection limits (DL) are presented in the Supplementary Materials (Table S1).

### 2.5. Meteorological and Air Quality Data

Meteorological data of the exposure period were obtained from the Barreiro-Lavradio meteorological station (38°40′28″ N, 9°2′51″ W) of Instituto Português do Mar e da Atmosfera (IPMA). Figure S1 (Supplementary Materials) provides an overview of the meteorological conditions during the exposure period. The mean daily temperature was 16.7 ± 3.2 °C (ranging from 9.7 to 25.1 °C), the mean daily relative humidity was 80.1 ± 7.9% (63.0 to 98.0%), the mean wind speed was 2.9 ± 1.0 m·s^−1^ (1.0 to 6.8 m·s^−1^), and the mean daily precipitation was 6.1 ± 7.2 mm (0.1 to 21.4 mm), in 41 days with precipitation. Since strawberry leaves are sensitive to wash-off [4], the effect of rain was limited by collecting the leaf samples a week after the last rain event (which was residual, 0.7 mm), as recommended in other studies [33]. The maximum wind speed was 3.9 m·s^−1^ in the last week of exposure and resuspension of PM caused by wind of 5 m·s^−1^ is considered small compared with total deposition on leaves [34]. During the exposure period, the prevailing wind directions were north (N), northwest (NW), and west (W) [26].

PM_2.5_ and PM_10_ concentrations from the Paio Pires monitoring station (38°37′16″ N, 9°4′55″ W) of the National Air Quality Monitoring Network were obtained through the information system QualAr [35].

### 2.6. Data Analysis

#### 2.6.1. Statistical Analysis

The Shapiro–Wilk test was performed to assess the normality of the variable distributions. A *t*-test and a non-parametric Wilcoxon Signed Rank test were performed (for normally and non-normally distributed data, respectively), to compare the elemental concentrations in the strawberry leaves exposed in the study area and at the background site, and also to compare the enrichment factors in the strawberry leaves and in the lichens simultaneously exposed. The Kruskal–Wallis tests were used to compare more than two independent groups. Spearman’s rank correlation was applied to assess the associations between element concentrations. The significance level was based on an alpha of 0.05.

The principal component analysis (PCA) was applied to explore the common contribution of different elements in the leaves. As it is not possible to consider all the variables in the analysis given the number of available cases (exposure sites), variables that had low values (r < 0.5) in the diagonal of the anti-image correlation matrix were eliminated from the analysis to obtain a significant result for Bartlett’s sphericity test (*p*-value < 0.001). The criterion of Kaiser–Meyer–Olkin (KMO) was 0.642, which confirmed the suitability of the data for PCA. The components with eigenvalues higher than 1 were considered for varimax rotation with Kaiser normalization to obtain the final matrix [36]. Factor loadings higher than 0.5 were considered to relate an element to a source [37]. Statistical analysis was performed using IBM SPSS Statistics 27 software.

For the comparison between biomonitors, the elemental concentrations in *Fragaria × ananassa* Duchesne ex Rozier leaves and in lichen *Flavoparmelia caperata* (L.) Hale samples were compared by means of major axis regression analysis, since an independent variable cannot be distinguished. In this situation, Model II regression is recommended for use instead of the usual Model I [38,39,40]. To conclude if there are differences in bioaccumulation between the species, a permutation test is performed to assess if the slope of the regression line that relates the concentrations in *Flavoparmelia caperata* (considered a good bioaccumulator) and *Fragaria × ananassa* is significantly different from 1 [40]. “R” package “lmodel2” was used to calculate the regression [41].

#### 2.6.2. Accumulation and Enrichment Factors

The Accumulation Factor (AF) was determined, for each sample, as the ratio of element concentrations (normalized to Al) in the strawberry leaves from the sampling site versus the leaves from the background site [42,43,44]:

(1) $${AF}_{X}=\frac{\left(\frac{C_{X}}{C_{Al}}\right)leaves\;sampling\;site}{\left(\frac{C_{X}}{C_{Al}}\right)leaves\;background\;site}$$

where C_X_/C_Al_ is the ratio between the element and Al concentrations in strawberry leaves. Al was used as the reference element [42,44], since it is considered a non-anthropogenic soil element. This was confirmed by the PM_2.5_ source apportionment by Positive Matrix Factorization (PMF) performed in the study area [27], which identified a “soil” emission source predominantly characterized by Al. The following contamination categories can be defined based on AF values [43]: AF ≤ 1 denotes no accumulation relative to background; 1 < AF < 2 minimal accumulation; AF= 2–5 moderate accumulation; AF = 5–20 significant accumulation; AF = 20–40 very high accumulation; AF > 40 extremely high accumulation.

The enrichment factor (EF) assists in identifying the crustal versus anthropogenic origin of elements and was determined for each sample [8,38,45]:

(2) $${EF}_{X}=\frac{\left(\frac{C_{X}}{C_{Al}}\right)sample}{\left(\frac{C_{X}}{C_{Al}}\right)crust}\;$$

where (C_X_/C_Al_)_sample_ is the concentration ratio of an element to Al in the exposed strawberry leaves (or lichens), and (C_X_/C_Al_)_crust_ is the corresponding ratio in the crust. The upper continental crustal average [46] was used. An EF lower than 10 is generically considered to indicate crustal origin, while an EF ≥ 10 indicates an element mostly emitted from anthropogenic sources [13,37]. According to [47], elements can be assumed to be enriched when the mean EF is larger than 3, with at least 30% of the samples with EF > 3.

#### 2.6.3. Distribution Maps of the Elemental Concentrations

Geostatistical modeling maps of elemental concentrations in strawberry leaves were prepared in QGIS version 3.28.6-Firenze (http://qgis.org), using the Inverse Distance Weighting (IDW) interpolation, as applied in other biomonitoring studies [11,26,48,49,50,51]. IDW calculates a cell value, averaging the sampled data around that cell [52]. In the equation for IDW (Equation (3)),

(3) $$v_{0}=\frac{\sum_{i=1}^{S}v_{i}\left(\frac{1}{d_{i}^{k}}\right)}{\sum_{i=1}^{S}\left(\frac{1}{d_{i}^{k}}\right)}$$

where v_0_ and v_i_ represent the estimated and the sampled value at point I, respectively, d_i_ is the distance to point I, S is the number of sampled points used in the calculation (equal to 47), and k is assumed equal to 2. The range of concentration values for each element was subdivided in intervals corresponding to 0–20, 20–40, 40–60, 60–80, and 80–100 percentiles. The values below detection for Cr and Pb were set to half of the detection limit for representation [53].

## 3. Results and Discussion

### 3.1. PM Levels

Figure S2 presents the variability of PM_2.5_ and PM_10_ levels during the exposure period, when a mean daily PM_10_ concentration of 19.3 ± 9.0 μg.m^−3^ (from 3.8 to 51.7 μg.m^−3^) was registered and the WHO daily guideline value for PM_10_ of 45 μg.m^−3^, not to be exceeded more than 3–4 days per year [54], has been exceeded three times. During the exposure period, a mean daily PM_2.5_ concentration of 9.5 ± 5.3 μg.m^−3^ (from 1.7 to 25.7 μg.m^−3^) was registered and the WHO daily guideline value for PM_2.5_ of 15 μg.m^−3^, not to be exceeded more than 3–4 days per year [54], has been exceeded 19 times.

The lower PM levels observed in 2020, and specifically in the strawberry plants exposure period, are attributable to the national lockdown decreed on 18 March until 4 May and other measures applied in the following months to slow down the transmission of SARS-CoV-2 [55], which have significantly impacted the anthropogenic activities in the study area, and consequently the PM levels, PM_2.5_ chemical composition, and source contributions [27]. Nevertheless, the industrial activity in the area did not stop during this period, creating an opportunity to assess the impact of industry on the local air quality.

During the exposure period, PM_10_ and PM_2.5_ concentrations were negatively correlated with precipitation (r = −0.485 and −0.487, respectively), as expected due to atmospheric washout, and also with mean daily wind speed (r = −0.333 and −0.489, respectively), since higher wind speeds promote the pollutants’ dispersion [4].

### 3.2. Elemental Characterization of Strawberry Leaves

The percentage of samples that could be recovered after exposure was 62.8%, a value similar to that obtained in the city of Antwerp (69.2%), although in the other cities of the StrawbAIRies project, much higher recoveries were obtained (globally, 88.7%) [22].

The descriptive analysis of elemental concentrations in the strawberry leaves is presented in Table 1 and Figure S3, where the elements are categorized in macro- and microelements, most of which are plant nutrients, and trace elements, which on the contrary, do not display a physiological function (namely Rb, Sr, Ba, Pb, Cr, As, and Br) [56].

Major elements measured in the strawberry leaves were the macronutrients and structural elements (K, Ca, Mg, P, S, Si), with mean concentrations ranging from 1.1 ± 0.4 to 24.5 ± 7.7 g.kg^−1^, while the micronutrients (Fe, Mn, Zn, and Cu) had concentrations ranging from 5.6 ± 1.6 to 2420 ± 1770 mg·kg^−1^ and trace elements, from 6.0 ± 2.5 to 108 ± 29 mg·kg^−1^. A study conducted in Antwerp (Belgium) found elemental concentrations in the surface of strawberry leaves assessed by ED-XRF ranging from 8.4 ± 3.0 ng.cm^−2^ (Cr) to 44,656 ± 9160 ng.cm^−2^ (Ca), with the following order: Cr < Pb < Rb < Ti < Sr < S < Fe < Si < P < Cl < Ca [4], which coincides generically with the results of the present study.

Markert [57] proposed the use of a “reference plant” with an average chemical composition. Several elements in the exposed strawberry leaves, namely all the non-essential trace elements (except for Rb) and Fe, presented mean concentration values above the “reference plant” levels [57]. The values are within the typical range found in terrestrial plants, except for K, Mg, P, S, Sr, Ba, Se, and Cr, for which above-normal concentrations were observed (Table 1). The toxic levels for plants were even reached for Sr and Cr. Although there is no consensus, 0.5–10 mg·kg^−1^ is indicated as a normal level of Pb in plants [62] and this value has been exceeded in some samples.

The elemental concentrations in the exposed leaves in the exposure sites were significantly higher (*t*-test and Wilcoxon test, *p* < 0.05) than in the background site, except for S, Br, and Se, for which the opposite was observed. For Cl, Mn, and Pb, a non-significant difference was found (Table 1). Although the reference site is located in a large natural or seminatural area and it was assumed as a background site, it is a weekend residential area, which may be affected by biomass burning emissions for household heating. The enrichment in S [69] and Br [70] may reflect these emissions. Furthermore, as Tomašević et al. [5] noted, the green areas near urban zones may not be true background sites, due to the transport of air pollutants.

**Figure 2:**
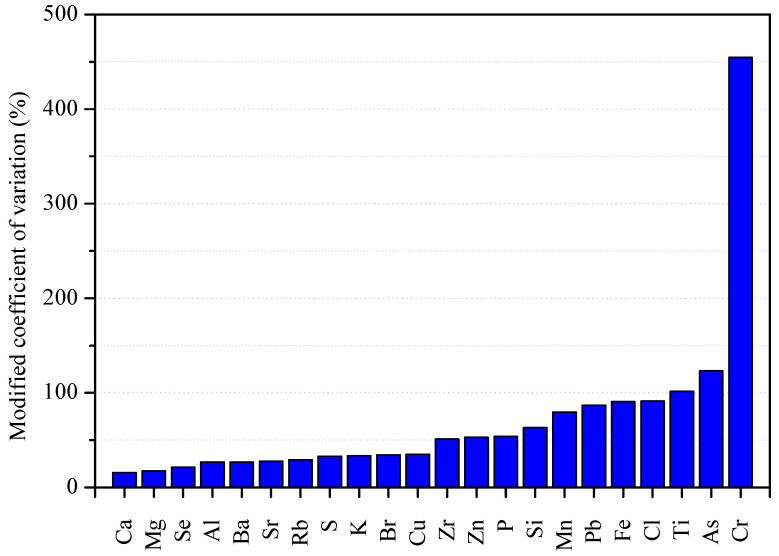
Modified coefficient of variation (standard deviation divided by the median) of element concentrations in strawberry leaves.

Not only high concentration, but also large element variability, indicate the influence of an anthropogenic source [71,72]. The modified coefficient of variation of element concentrations (standard deviation divided by the median) was determined [15], confirming the existence of high differences in concentration and dispersion between elements (Figure 2). Most macro- and micronutrients (Ca, Mg, S, K, P, Cu, and Zn) presented a low coefficient of variation, suggesting that these elements are regulated by biological processes, whereas Fe and Mn, despite being micronutrients, presented higher values. As generically observed for non-essential trace elements [56,71], Pb, Ti, As, and especially Cr presented higher spatial variability.

### 3.3. Assessment of Pollution Sources

#### 3.3.1. Bioaccumulation

**Figure 3:**
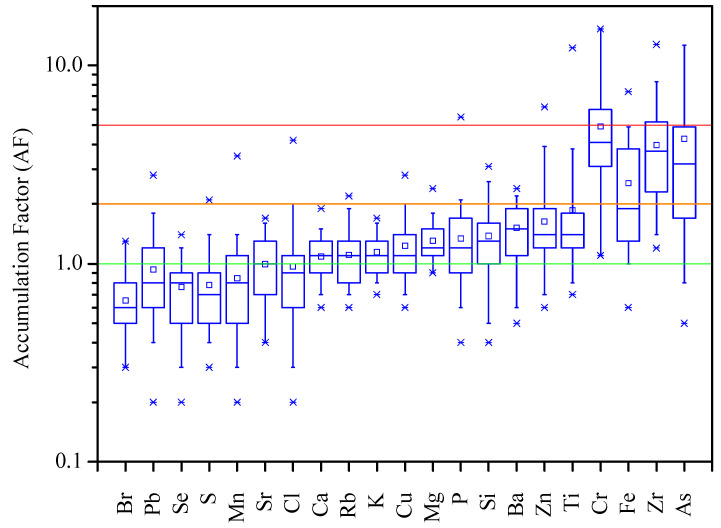
Accumulation Factor (AF) of the elements in exposed strawberry leaves. In the box plot, the square represents the mean, upper and lower times sign (×) represent the maximum and minimum values, and the whiskers extend to 1.5* the interquartile range. Below green line—no accumulation; green to orange line—minimal accumulation; orange to red line—moderate accumulation; above red line—significant accumulation.

As previously mentioned, elements with an Accumulation Factor (AF) higher than 2 are considered to evidence enrichment and environmental pollution [43]. Figure 3 presents the AF for the different chemical elements in the exposed strawberry leaves. The obtained AF values reflect plant biochemistry, since all plant macronutrients (K, Mg, Ca, P, and S) and some micronutrients (Mn, Cu, and Cl) presented AF < 1.5. Furthermore, results indicate a minimal accumulation of Ba (1.5 ± 0.5), Zn (1.6 ± 1.0), and Ti (1.9 ± 1.9), and moderate accumulation of Fe (2.5 ± 1.5), Zr (4.0 ± 2.3), As (4.3 ± 4.7), and Cr (4.9 ± 3.5). No accumulation of Br (0.7 ± 0.2), Se (0.8 ± 0.3), Pb (0.9 ± 0.5), and Sr (1.0 ± 0.4) was found, probably due to environmental contamination on the background site. Although unexpected, other works found Pb contamination even in remote areas [13].

A non-normalized AF with a value of 3 for Fe was also observed to arise from PM contamination from steel industry in other works [73]. An AF of 3.55 for Cr was observed in the *Quercus ilex* L. leaves and was considered to result from the uptake from the air [74]. Castanheiro et al. [4] also identified a significant accumulation of Cr in strawberry leaves exposed in a suburban site, and this was attributed to traffic PM.

**Figure 4:**
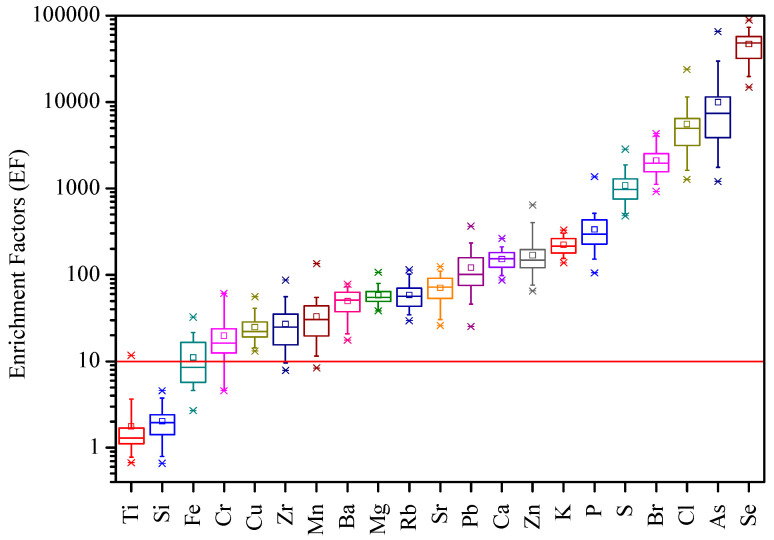
Enrichment factor (EF) of the elements in exposed strawberry leaves. Above the red line, anthropogenic sources are present.

To discriminate between anthropogenic and natural sources of the elements measured in the strawberry leaves, enrichment factors (EFs) relative to average crustal composition were determined, considering Al as the normalizing element (Figure 4). The elements Ti (1.8 ± 1.8) and Si (2.0 ± 0.9) have a predominant crustal origin (EF < 10), while Fe (11.1 ± 6.5), Cr (19.7 ± 14.1), and the remaining elements showed the contribution of anthropogenic emissions.

In this work, it is not possible to quantify the washout effect by precipitation (which was residual in the last week of exposure) and by quickly rinsing the leaves in the samples preparation, but a part of the PM may have been removed from the leaves’ surfaces by those processes [4]. As such, the enrichment results determined in this work may be underestimated for some elements.

#### 3.3.2. Spearman Correlations

Spearman correlations were determined to assess the bivariate relations between elemental concentrations in the exposed strawberry leaves, and significant correlations are presented in Table S2. Fe was strongly correlated with Cr (r = 0.831), and Cr was also moderately correlated with Mn (r = 0.605). This suggests that these elements have a common emission source, responsible for the observed EF ≥ 10 for these elements, which probably is the steel industry. This corroborates the findings of the biomonitoring study with lichens in the study area [26], where the strong correlations found between Fe-Cr (r = 0.81), Cr-Mn (r = 0.92), and Fe-Mn (r = 0.77) were assigned to the steelworks. In the strawberry leaves, the Cr-Mn (r = 0.605) and Fe-Mn (r = 0.336) correlations, although significant, were not as strong as in lichens, indicating the presence of mixed sources, namely the root uptake of Mn as a minor nutrient of plants.

In the strawberry leaves, Fe was also strongly correlated with Ti (r = 0.742) and significantly correlated with Al (r = 0.493), all elements of geogenic dust [72,75]. This mixed natural and industrial influence was previously found for Fe in other studies near steelworks [76] and also in the biomonitoring study with lichens in the study area [26], where Fe-Ti (r = 0.84) and Fe-Al (r = 0.87) correlations were found. Differently from our study, the enrichment in Cr and Fe in strawberry leaves has been related to traffic in a Belgian urban area [4].

While Zn and Pb are frequently associated with the steel industry [77,78], in the present study these elements seem to be associated with a distinct emission source, namely traffic, since their correlation with Fe, Cr, and Mn is not significant. Castanheiro et al. [4] also observed the accumulation of Pb in strawberry leaves, which was attributed to traffic. The pair Zn-Pb has been reported to present antagonistic interaction in plant physiology, with competitive inhibition by one another [8,68]. It is thus plausible that the weak Zn-Pb correlation (r = 0.460) observed in strawberry leaves is influenced by this physiological interaction.

Additionally, another anthropogenic source is suggested by the moderate correlation between As and Zr (r = 0.686). Since P and S are major nutrients for plant growth, the significant although weak correlations between As/Zr with P and S may be due to root absorption. Moderate correlations were found between K-Al (r = 0.682), K-Cl (r = 0.648), Cl-Si (r = 0.616), and Cl-Al (r = 0.586), suggesting the geogenic nature of K and Cl. Although Sr is not essential for plants, it is absorbed from the soil along with Ca [66], and this might explain the Sr-Ca correlation (r = 0.590).

#### 3.3.3. Principal Component Analysis

Principal component analysis (PCA) was performed to estimate the profiles of possible emission sources, as previously performed using the concentration of trace elements in leaves [8,37,42,43,66]. Table 2 presents the PCA results for the element concentrations in the exposed strawberry leaves.

The four most discriminant components—PC1 to PC4 (Table 2)—accounted for 71.7% of the total variance in all exposed strawberry leaves. PC1 explained 29.2% of the variability and had high positive loadings for K, Al, Si, Cl, and Mg, which are crustal and leaf-occurring macro-elements. PC2 explained 18.8% of the variability and included Cr, Fe, and Mn, which are tracers of the iron and steel industry [70], and a non-negligible contribution of Ti. PC3 explained 14.3% of the variability and had higher loadings of P, S, and Mg, which are plant major nutrients, absorbed by root uptake. PC4 explains 9.4% of the variability and includes Zn, Pb, and Ti, probably denoting a traffic origin, with resuspension. In fact, Zn and Pb concentrations in tree leaves have been associated with traffic [43,79], since Zn is emitted by the abrasion of tires and brakes, and from lubricating oils, and Pb is emitted by exhaust gases and brakes [16]. Despite the removal of leaded fuels many years ago, Pb is a still traffic-related element that has been observed with several biomonitors [4,11,37,71,80]. The resuspension of contaminated soil can act as a source of Pb, as is probably the case, given the significant contribution of Ti for PC4.

A source apportionment of PM_2.5_ was also performed in the study area in 2020 [27], where a total of seven emission sources were identified by the Positive Matrix Factorization (PMF) receptor model: soil (defined by Al, Si and Ti), sea (Na, Cl and Mg), secondary sulphate (SO_4_^2−^, NH_4_^+^), industry/steelworks (Fe, Mn and Ca), vehicle non-exhaust (Zn, Pb, Mn, Cu, and Fe), vehicle exhaust (BC, NO_3_^−^, and NH_4_^+^) and fuel-oil combustion (V, K). It is possible to conclude that the biomonitoring strategy with strawberry leaves reflected some of these PM_2.5_ emission sources, namely, soil/geogenic, industry, and traffic.

#### 3.3.4. Spatial Distribution of Elemental Concentrations

**Figure 5:**
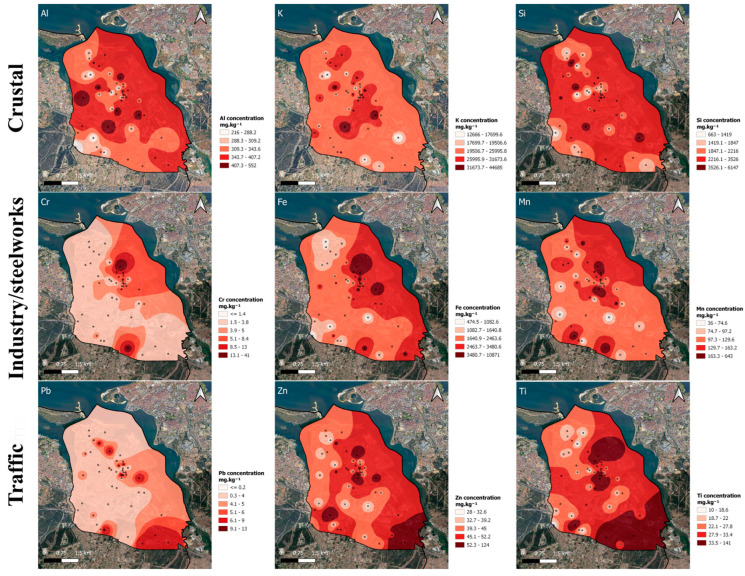
Spatial distribution of element concentrations in the exposed strawberry leaves in the study area (in mg·kg^−1^) related to (**above**) crustal natural origin, (**middle**) industry/steelworks, and (**below**) traffic.

The distribution maps of the element concentrations in the exposed strawberry leaves were created (Figure 5). The similarities between the spatial distributions obtained for the elements grouped by PCA support the contribution of the identified emission sources. Maximum concentrations of Cr, Fe, and Mn were obtained near the steelworks, with isolated hotspots in the south of the grid, while the maximum concentrations of Zn and Pb were obtained in the south, close to the highways, with isolated hotspots in the industrial area.

**Figure 6:**
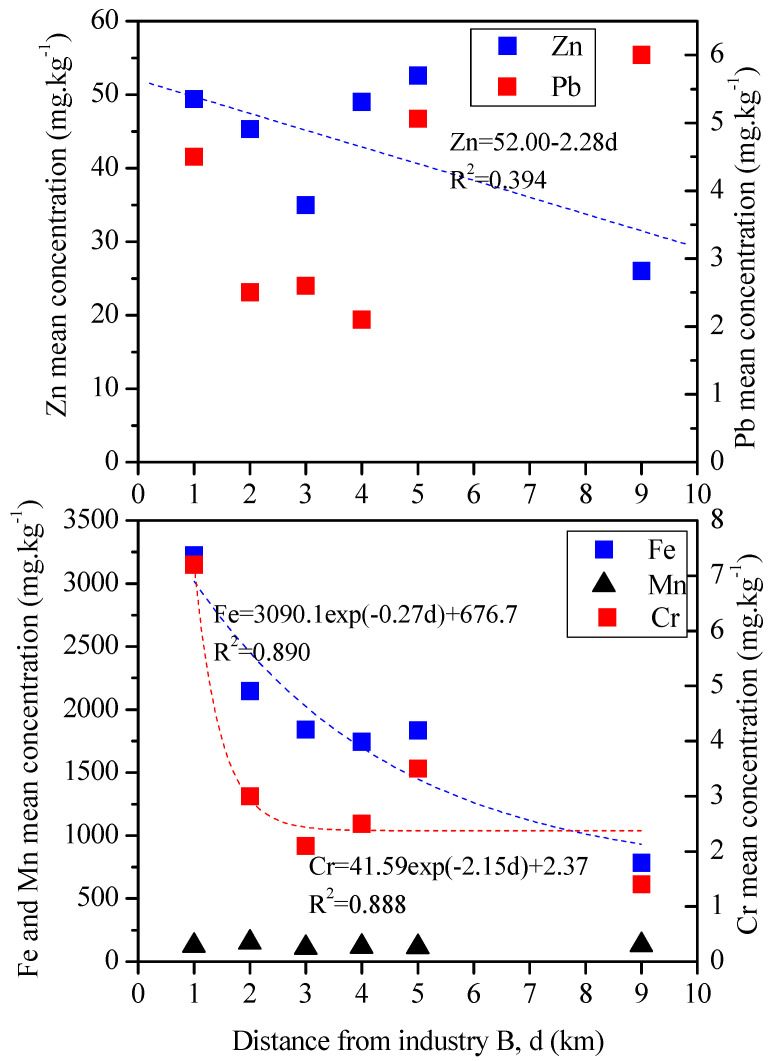
Mean elemental concentrations found in the exposed strawberry leaves versus the distance to steelworks B.

To confirm the influence of the steelworks in the local air quality, the study area was divided into concentric crowns with a radius of 1 to 5 km, with the center in steelworks B. Figure 6 presents mean element concentrations found in the exposed strawberry leaves versus the distance to steelworks B. Fe and Cr concentrations declined exponentially with distance from the source, according to strong models (r^2^ = 0.890 and 0.888 for Fe and Cr, respectively). The decrease in metal content in leaves with increasing distance from metallurgic industries was identified in other studies [14,15]. The peak levels of Fe and Cr immediately around the steelworks may be due to fugitive emissions from stockpiles, loading/unloading operations, and heavy-duty traffic.

Despite the observed relation of Mn with steelworks emissions, Mn concentrations are not significantly higher near the steelworks. Lower Mn concentrations in leaf samples at a Polish polluted site compared to others have been identified [81], and a decrease in Mn concentration in birch leaves near a smelter due to SO_2_ emissions has also been reported [82]. The concentrations of Pb, Zn, and also Cr increased at a distance of 4–5 km from steelworks B, which is consistent with the influence of the highway traffic.

Figure S4 shows the variability of Fe levels considering the distance from steelworks B, divided by quadrants. The southeast (SE) quadrant presented significantly higher Fe concentrations than the NW and SW quadrants, according to the Kruskal–Wallis test (*p*-value = 0.004 and 0.039, respectively). This fact may be due to the influence of the prevailing wind directions, which were mostly from the north (N), northwest (NW), and west (W) in the sampling period [26].

### 3.4. Comparison Between Biomonitors

As stated before, for each potted strawberry plant that was exposed, a transplanted lichen (*Flavoparmelia caperata* (L.) Hale) sample was exposed simultaneously, and the results of the biomonitoring study using lichens have been described in detail elsewhere [26]. This strategy may provide insights about whether the use of strawberry leaves as a biomonitor of air pollution affords similar results as lichens. For this, a similar data analysis to that conducted for the strawberry leaves is now performed for lichens, namely the assessment of EFs and PCA.

#### 3.4.1. Enrichment Factors

Figure S5 presents the mean EFs assessed in the strawberry leaves and lichens simultaneously exposed in the study area. Regarding the EFs found in lichens using the reference soil composition and Si as the normalizing element, as assessed by Abecasis et al. [26], Al and Mg were found to have a predominant crustal origin (EF < 10), while Fe (10.5 ± 2.3), Ti (11.7 ± 1.6), and the remaining elements were found to show anthropogenic contributions. Nevertheless, when the EF in lichens is determined using Al as the normalizing element as presented in Figure S5 (same methodology as that used for strawberry leaves), Fe (7.3 ± 1.6) and Ti (8.2 ± 1.1) present an EF below 10, suggesting also the importance of soil origin for these elements.

Overall, comparing the mean EF values in the strawberry leaves and in lichens, both determined using Al as the normalizing element (Figure S5), it can be concluded that strawberry leaves are significantly more enriched than lichens in Si, Fe, Zr, Mn, Rb, Sr, S, As, and especially Mg, K, and Cl (*p*-value < 0.001), and lichens are more enriched than strawberry leaves in Ti, Cu, Pb, Ca, Zn (*p*-value < 0.001), and Br (*p*-value = 0.025), as assessed by the Wilcoxon test. The differences between the two biomonitors in the case of Cr and Se are not significant (*p*-value > 0.05). The different morphological and physiological characteristics (such as growth rates) of the two biomonitors play a role in the different accumulation capabilities, but the higher enrichment of strawberry leaves in plant macro- and micronutrients (K, Mg, S, and Mn) may also be due to root absorption.

Major axis regression analysis was used for comparison of the two co-located biomonitors, as described elsewhere [38,40]. The major axis regressions of the EFs were significant for Fe (*p*-value = 0.017), Zn (*p*-value = 0.001), Pb (*p*-value = 0.024), and Ti (*p*-value = 0.008), confirming that *Fragaria × ananassa* Duchesne ex Rozier is a useful biomonitor of air pollution associated with these elements. Slope values were nevertheless all significantly different from 1 (permutation test *p*-value < 0.050). No significant regression could be found for Cr (*p*-value > 0.050), probably due to the number of data points below DL, in lichens and/or strawberry leaves. The absence of significant regression for the remaining elements reflects that the two species must take up these elements in different ways, with a component of root uptake in the case of strawberry plants, as expected for K, Ca, Mg, S, Mn, Cu, Cl, and Si, which are nutrients or structural elements in plants.

It is not possible in our study to determine exactly what proportion of trace elements was taken up from the soil and what came from atmospheric deposition on leaves. The soil can contribute to Zn and Cu in leaves [74], as these elements are characterized by easy translocation to leaves [14]. On the other hand, the uptake of Pb and As from soil is low [10,11,43,83], the translocation of Pb from roots to leaves is limited [14,74], and thus Pb in leaves is considered to come mostly from atmospheric deposition [59,84]. Fe has very low mobility from soil [11]. Fe and Cr tend to accumulate in roots and are scarcely translocated into leaves [85] (and references therein), and hence, the observed leaf accumulation of these metals appears to be mainly due to atmospheric deposition. Moreover, Fe was already identified as a good indicator for atmospheric PM on leaves [86].

#### 3.4.2. Principal Component Analysis

PCA was performed on the element concentrations in the lichens simultaneously exposed in the study area. Table 2 (above) provides the description of the three identified factors, for the chemical species quantified in the lichens. The same criteria for the selection of variables were adopted for strawberry leaves and lichens, except that for lichens, a low value (r = 0.324) for Pb was retained in the diagonal of the anti-image correlation matrix, affording a significant result for Bartlett’s test (*p*-value < 0.001) and a Kaiser–Meyer–Olkin criterion of 0.737, confirming the suitability of the data for PCA.

The three most discriminant components (PC1, PC2, and PC3) account for 75.4% of the total variance in the lichens’ concentrations. PC1 explained 46.5% of the variability and had high positive loadings for Mn, Cr, Fe, and Mg. As in the strawberry leaves, this factor can be assigned to the steelworks’ emissions, with resuspension suggested by the contribution of the crustal element Mg. This component had a non-negligible loading for Zn, and in fact, Zn is also considered a steel industry tracer [70]. PC2 explained 19.3% of the variability and had high loadings in Si, Al, Ti, and K, and a non-negligible loading in Fe, all crustal elements. PC3 explained 9.6% of the variability and had higher loadings in S, Zn, and Pb, being assigned to traffic, since namely S is considered a tracer of diesel exhaust [70].

Overall, it can be concluded that there is a similar result regarding the identification of pollution sources, since a parallel was found between the principal factors contributing to the element concentrations in strawberry leaves and lichens, with the exception of plant major nutrients component (by root uptake), which is not present in lichens.

## 4. Conclusions

This study aimed to identify the main pollution sources and the hotspots of air pollution in an urban-industrial area (Seixal, Portugal), applying a strategy based on a biomonitoring study of air pollution, using strawberry plant (*Fragaria × ananassa* Duchesne ex Rozier) leaves, where the locations of the exposed biomonitors were defined by the participation of citizens, within a citizen science project.

Strawberry leaves were found to be highly enriched in all elements, except Ti, Si, and Al, and some samples even reached phytotoxic levels for Sr and Cr. Accumulation relative to a reference site was moderate for Cr, Fe, Zr, and As, and minimal for Ba, Zn, and Ti.

Principal component analysis was performed and allowed us to discriminate a crustal source (Al, Si, K, Cl, and Mg), a source of plant major nutrients absorbed by root uptake (P, S, and Mg), and two anthropogenic sources, one associated with steelworks (Cr, Fe, and Mn) and the other with traffic dust resuspension (Zn, Pb, and Ti). Element spatial distribution allowed us to confirm these pollution sources. Exponential decreasing trends of Fe and Cr concentrations with the distance from steelworks were also identified, along with maximum concentrations of Zn and Pb close to highways, indicating a good biological response of strawberry leaves to atmospheric pollution.

The comparison of the performance of strawberry leaves and lichens as biomonitors, carried out by regression analysis of EFs, showed a significant relation for Fe, Pb, Ti, and Zn, confirming that strawberry leaves are a useful biomonitor of air pollution associated with these elements. Furthermore, PCA applied to both types of biomonitors identified the same three pollution sources—crustal, steelworks, and traffic.

Overall, the use of strawberry plant leaves in biomonitoring studies, following a citizen science approach, was confirmed to provide information on the main sources and hotspots of air pollution in the area, allowing us to involve the local community.

## Figures and Tables

**Table 1:** Descriptive statistics of the element concentrations (in mg·kg^−1^, dry weight) in the strawberry leaves (n is the number of exposed samples in which the element concentration was above the DL and SD stands for standard deviation). Comparison with reference plant, typical concentrations, and toxic levels for terrestrial plants, and with the mean element concentrations found in lichens simultaneously exposed in the study area. Significant differences in the element concentrations between exposed strawberry leaves in the exposure sites and in the background site were assessed by *t*-test and Wilcoxon test (for variables with normal and non-normal distributions, respectively), at α = 0.050.

Strawberry Leaves (Present Study)								Reference Plant [57]	Typical Concentrations in Plants	Toxic Levels in Plants	Transplanted Lichens [26]
Element	n	Mean ± SD	Median	Min	Max	Background Site	*p*-Value				Mean ± SD
		mg·kg^−1^						mg·kg^−1^			
**Macro elements**											
K	47	24,500 ± 7700	23,400	12,700	44,700	18,000	0.000	19,000	3500–6600 ^n^		7900 ± 1120
Ca	47	22,600 ± 3600	23,000	11,100	30,200	18,200	0.000	10,000	2300–5000 ^n^		226,000 ± 15,000
Mg	47	4970 ± 840	4970	3390	7330	3320	0.000	2000	500–1300 ^n^		1230 ± 270
Si	47	2400 ± 1260	1960	660	6150	1450	0.000	1000			6240 ± 1280
P	47	1440 ± 680	1250	570	3900	940	0.000	2000	1100 ^n^		n.d.
S	47	1140 ± 380	1080	630	2170	1290	0.011	3000	10–150 ^a^		1580 ± 410
**Micro elements**											
Cl	47	3180 ± 2490	2710	480	14,600	2650	0.154	2000			n.d.
Fe	47	2420 ± 1770	2090	480	10,870	780	0.000	150	640–2486 ^b^; 42–352 ^i^		11,800 ± 3800
Al	47	347 ± 87	325	216	552	291	0.000	80	200–1000 ^b^; 10–900 ^i^		2590 ± 450
Mn	47	128 ± 90	111	36	643	132	0.756	200	15–100 ^b^; 15–160 ^i^		406 ± 297
Zn	47	47.3 ± 21.3	40.0	28.0	124.0	26.0	0.000	50	10–160 ^b^; 20–400 ^c^; 10–150 ^e,h^; 15–30 ^f,i^	80–200 ª; 70–400 ^b^; 100 ^c,f^; 300–400 ^e^	608 ± 454
Cu	46	5.6 ± 1.6	5.0	3.0	10.0	4.0	0.000	10	0.4–45.8 ^b^; 2–20 ^c,g^; 2–12 ^f^; 3–30 ^h^; 4–5 ^i^	100 ^a^; 20–30 ^c^; 20–100 ^e,h^; 30 ^f,g^	163 ± 90
**Trace elements**											
Sr	47	108 ± 29	109	31	160	96	0.007	50		30 ^m^	565 ± 74
Ba	47	86.2 ± 22.7	89.0	30.0	128.0	50.0	0.000	40	2–13 ^l^	200–500 ^l^	n.d.
As	47	69.8 ± 62.7	51.0	9.0	320.0	15.0	0.000	0.1			30 ± 28
Ti	47	31.6 ± 25.8	26.0	10.0	141.0	15.0	0.000	5			1150 ± 230
Br	47	21.4 ± 7.0	20.0	9.0	48.0	29.0	0.000	4			180 ± 16
Rb	47	21.4 ± 5.9	20.0	12.0	39.0	17.0	0.000	50			70 ± 15
Zr	47	17.9 ± 8.1	16.0	5.0	39.0	4.0	0.000	0.1			79 ± 20
Se	47	9.3 ± 2.2	10.0	3.0	13.0	11.0	0.000	0.02	0.05–1 ^k^		75 ± 6
Cr	18	9.2 ± 8.9	6.0	3.0	41.0	<DL	0.003	1.5	0.1–5 ^e^; 1–2 ^i^	5–10 ^a^; 5–30 ^e,j^	52 ± 91
Pb	28	6.0 ± 2.5	5.0	2.0	13.0	6.0	0.918	1	1–13 ^c^; 3.0–5.9 ^f^ 0.5–10 ^h^; 0.4–2.5 ^i^; 3–10 ^j^	30 ^a,f^; 20 ^b^; 10 ^c^; 3–20 ^d^; 30–300 ^e,h^	130 ± 111

^a^ [58]; ^b^ [42]; ^c^ [59]; ^d^ [43]; ^e^ [8]; ^f^ [60]; ^g^ [61]; ^h^ [62]; ^i^ [38]; ^j^ [63]; ^k^ [64]; ^l^ [65]; ^m^ [66]; ^n^ [67] and references therein, namely [68].

**Table 2:** PCA results for the element concentrations in strawberry leaves and in the simultaneously exposed lichens, with factor loadings of principal components (PCs) and communalities. Loadings higher than 0.5 are in bold.

Strawberry Leaves						Lichens				
	PC1	PC2	PC3	PC4	Communality		PC1	PC2	PC3	Communality
K	**0.85**	0.006	0.172	−0.106	0.764	Mn	**0.965**	0.088	0.145	0.96
Al	**0.83**	0.289	0.067	−0.058	0.78	Cr	**0.96**	0.068	0.185	0.96
Si	**0.78**	0.118	−0.296	−0.1	0.72	Fe	**0.856**	0.437	0.154	0.946
Cl	**0.645**	0.07	0.319	0.028	0.523	Mg	**0.74**	0.327	0.077	0.661
Cr	0.096	**0.941**	0.106	0.028	0.906	Si	0.277	**0.902**	0.005	0.891
Fe	0.205	**0.889**	0.048	0.203	0.876	Al	0.347	**0.871**	−0.026	0.88
Mn	0.024	**0.5**	0.249	0.034	0.314	Ti	0.259	**0.817**	0.027	0.735
P	0.029	0.22	**0.853**	−0.084	0.784	K	−0.422	**0.658**	0.12	0.625
S	0.141	0.215	**0.778**	−0.15	0.693	S	0.236	0.307	**0.734**	0.689
Mg	**0.583**	−0.2	**0.584**	0.096	0.731	Zn	0.474	0.044	**0.606**	0.594
Zn	−0.125	0.243	0.235	**0.831**	0.82	Pb	−0.034	−0.124	**0.579**	0.351
Pb	−0.098	−0.251	−0.366	**0.725**	0.731					
Ti	0.039	0.413	−0.25	**0.665**	0.677					

## Data Availability

The raw data supporting the conclusions of this article will be made available by the authors on request.

## Supplementary Materials

The following supporting information can be downloaded at: https://www.mdpi.com/article/10.3390/plants13243587/s1. Figure S1: Daily meteorological data during the exposure period of the strawberry plants; Figure S2: Daily PM_2.5_ and PM_10_ concentrations measured in the Paio Pires monitoring station during the exposure period; Figure S3: Descriptive statistics of the element concentrations in the exposed strawberry leaves; Figure S4: Mean Fe concentration in strawberry leaves along the distance from steelworks B in each quadrant, and mean concentration per quadrant; Figure S5: Mean enrichment factor (EF) in the strawberry leaves and lichens that were simultaneously exposed in the study area. Table S1: Instrumental detection limits and results of XRF analysis of plant reference samples PTNATIAEA/19 and PTNATIAEA/20; Table S2: Spearman correlation coefficients between element concentrations in the exposed strawberry leaves.
