# Supplementary material for: Strawberry Plant as a Biomonitor of Trace Metal Air Pollution—A Citizen Science Approach in an Urban-Industrial Area near Lisbon, Portugal
Source: Plants (Basel). 2024 Dec 23;13(24):3587. doi: 10.3390/plants13243587 (PMC11679698; doi:10.3390/plants13243587)
Supplement: Supplementary file 1 [file plants-13-03587-s001.zip › plants-3344151-supplementary.pdf]

Strawberry Plant as Biomonitor of Trace Metal air Pollution—A Citizen Science Approach in an Urban-Industrial Area near Lisbon, Portugal

Carla A. Gamelas [1,2,*], Nuno Canha [1,3,*], Ana R. Justino [1], Alexandra Nunes [1], Sandra Nunes [4,5], Isabel Dionísio [1], Zsofia Kertesz [6], S. Marta Almeida [1]

[1] Centro de Ciências e Tecnologias Nucleares, Instituto Superior Técnico, Universidade de Lisboa, Estrada Nacional 10, Km 139.7, 2695-066 Bobadela LRS, Portugal; smarta@ctn.tecnico.ulisboa.pt (S.M.A.)

[2] Instituto Politécnico de Setúbal, Escola Superior de Tecnologia de Setúbal, Campus do IPS, Estefanilha, 2914-508 Setúbal, Portugal

[3] Hylab—Green Hydrogen Collaborative Laboratory, Central Termoeléctrica, 7520-089 Sines, Portugal

[4] Instituto Politécnico de Setúbal, Escola Superior de Ciências Empresariais, Campus do IPS, Estefanilha, 2914-508 Setúbal, Portugal; sandra.nunes@esce.ips.pt

[5] Center for Mathematics and Applications, NOVAMATH, Universidade Nova de Lisboa, 2829-516 Caparica, Portugal

[6] HUN-REN Institute for Nuclear Research (ATOMKI), 4026 Debrecen, Hungary; zsofi@atomki.hu

* Correspondence carla.gamelas@ctn.tecnico.ulisboa.pt or carla.gamelas@estsetubal.ips.pt (C.A.G.); nuno.canha@hylab.pt (N.C.)

Supplementary Material

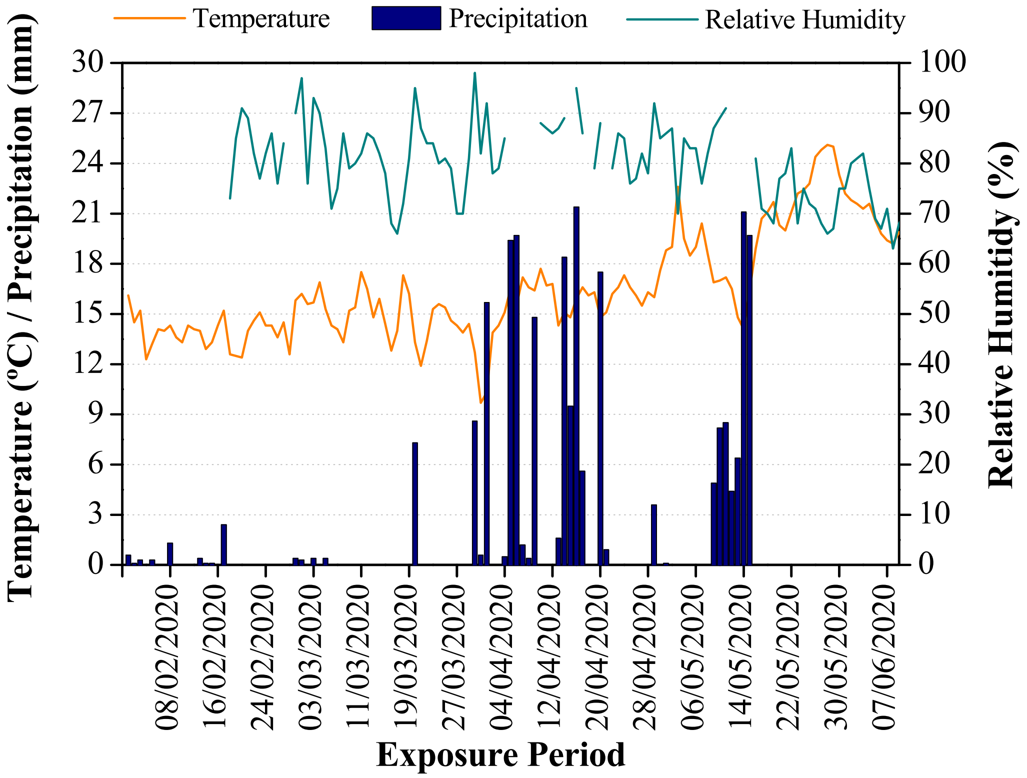

Figure S1. Daily meteorological data (temperature, precipitation and relative humidity) during the exposure period of the strawberry plants.

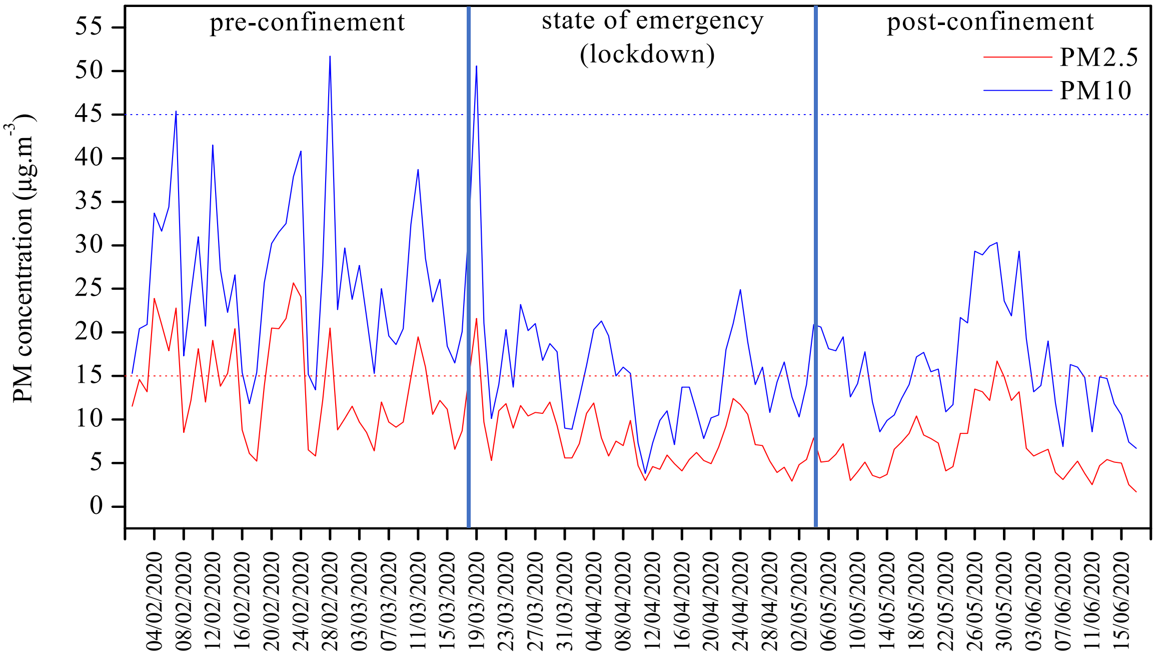

Figure S2. Daily $PM_{2.5}$ and PM_{10} concentrations measured in the Paio Pires monitoring station during the exposure period (specifying the COVID-19 lockdown period). Horizontal lines stand for the WHO daily guideline values for PM_{10} and $PM_{2.5}$.

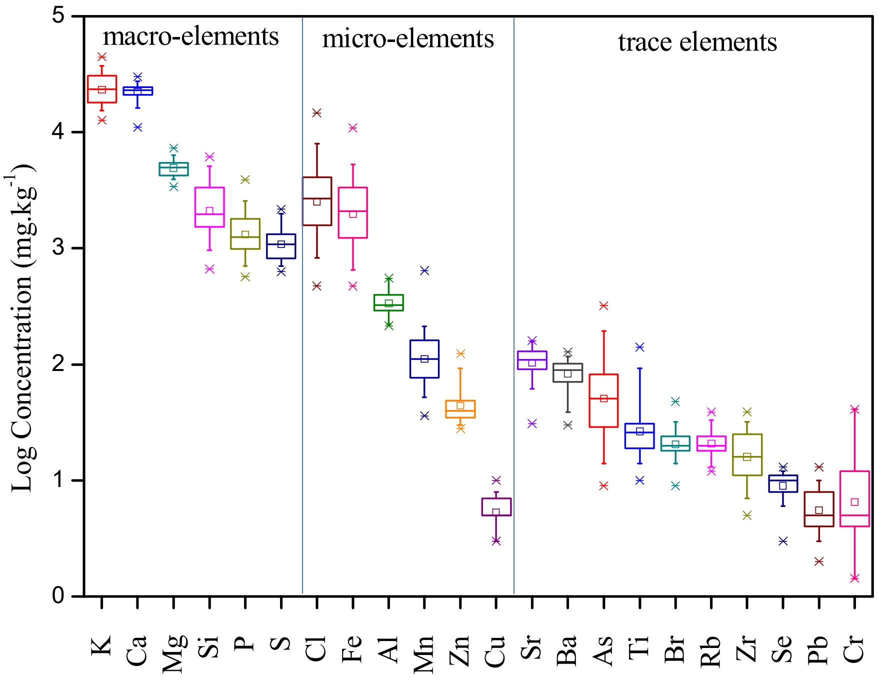

Figure S3. Descriptive statistics of the element concentrations in the exposed strawberry leaves. In the box plot, the square represents the mean, upper and lower times sign (x) shows the maximum and minimum values, the box provides the 25th percentile, the median and the 75th percentile, and the whiskers extend to 1.5* the interquartile range.

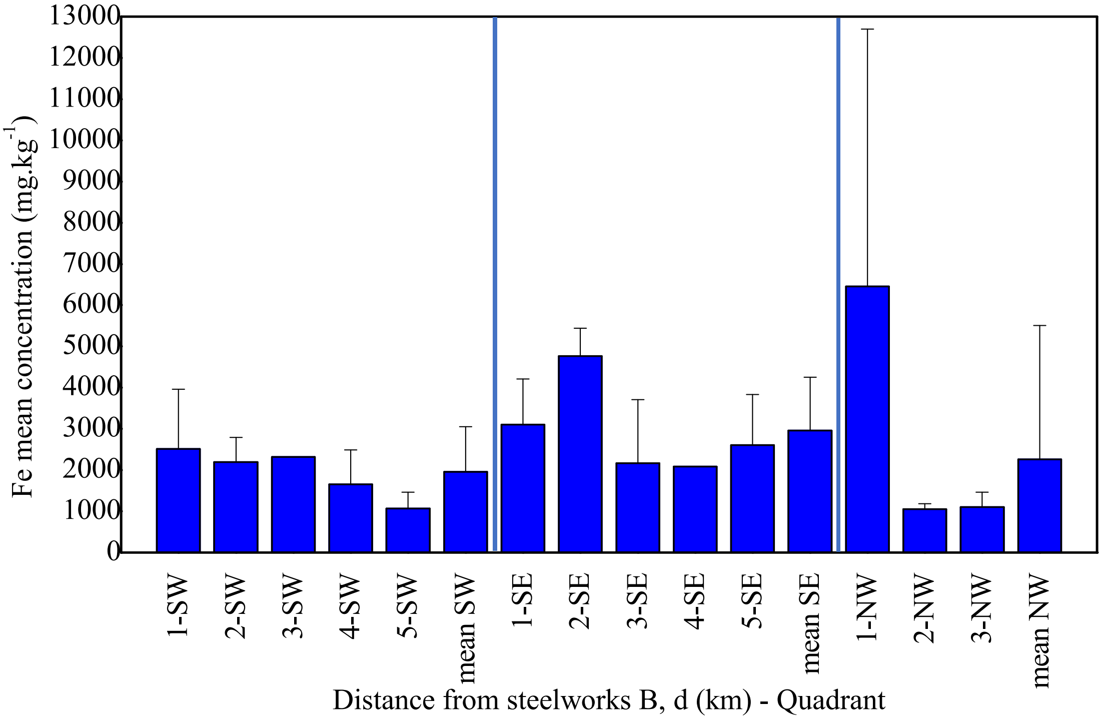

Figure S4. Mean Fe concentration in strawberry leaves along the distance from steelworks B (from 1 to 5 km) in each quadrant (SW, SE, NW), and mean concentration per quadrant

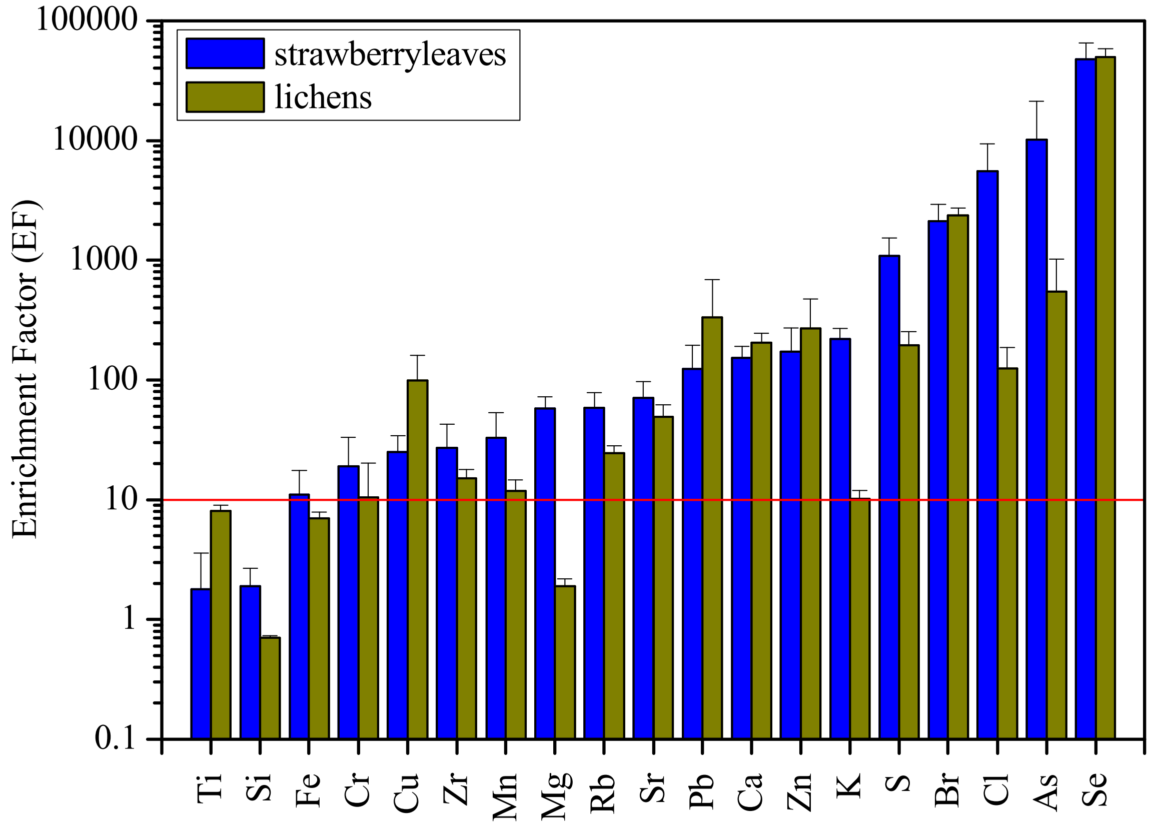

Figure S5. Mean Enrichment Factor (EF) and standard deviation, in the strawberry leaves and lichens that were simultaneously exposed in the study area, using Al as the normalizing element.

Table S1. Instrumental detection limits (DL) in mg.kg^{-1} and results of XRF analysis of plant reference samples PTNATIAEA/19 and PTNATIAEA/20 with standard deviation (SD)

	**PTNATIAEA/19**				**PTNATIAEA/20**				
	certified/ consensus value	SD	measured value	SD	certified/ consensus value	SD	measured value	SD	**DL**
	(mg.kg^{-1})				(mg.kg^{-1})				(mg.kg^{-1})
**Na**									93.4
**Mg**	2300	600	2360	635	1350	300	1920	863	167.3
**Al**									12.7
**Si**	460	200	590	271	350	200	315	189	32.9
**P**	2420	500	2240	282	4100	900	4610	2250	10.5
**S**	760	90	730	90	1060	200	935	154	9.0
**Cl**	214	40	260	17	388	170			5.4
**K**	11400	1400	11140	1588	3670	600	4339	2196	9.1
**Ca**	15600	3000	14980	2095	130	120	111	55	6.3
**Ti**	9	7	10	3					3.6
**Cr**									2.9
**Mn**	110	20	116	23	7	2	9	5	3.2
**Fe**	90	20	97	17	36	16	46	27	3.2
**Cu**	8	3	8	2	4.5	3	4	3	1.0
**Zn**	33	7	36	8	24	6	46	15	1.9
**As**									1.9
**Se**									0.3
**Br**									0.8
**Rb**	11	2	11	2					1.0
**Sr**	38	6	28	2					2.6
**Zr**									1.7
**Ba**									2.0
**Pb**									0.3

Table S2. Spearman correlation coefficients between element concentrations in the exposed strawberry leaves, significant at *p < 0.050 and **p < 0.010. Values in bold represent strong (r ≥ 0.70) and medium correlations (0.50 - 0.70).

	**Mg**	**Al**	**Si**	**P**	**S**	**Cl**	**K**	**Ca**	**Ti**	**Cr**	**Mn**	**Fe**	**Cu**	**Zn**	**As**	**Se**	**Br**	**Rb**	**Sr**	**Zr**	**Ba**	**Pb**
**Mg**	--																					
**Al**	.395**	--																				
**Si**	.333*	.448**	--																			
**P**	.416**			--																		
**S**	.453**			.486**	--																	
**Cl**	.385**	**.586****	**.616****			--																
**K**	.424**	**.682****	.438**			**.648****	--															
**Ca**	.346*		.428**	-.302*		.299*		--														
**Ti**		.379**	.378**		-.325*				--													
**Cr**									**.572***	--												
**Mn**										**.605****	--											
**Fe**		.493**							**.742****	**.831****	.336*	--										
**Cu**												.455**	--									
**Zn**			-.305*	.323*					.496**				.398**	--								
**As**				.396**	.356*										--							
**Se**	-.436**	-.503**	-.334*	-.316*		-.608**	-.631**									--						
**Br**					-.364*	.410**									**-.617****		--					
**Rb**							.458**											--				
**Sr**				-.370*	-.369*			**.590****							**-.764****	.330*	.448**		--			
**Zr**				.449**	.314*										**.686****		-.434**		**-.555****	--		
**Ba**						-.310*		.344*						-.311*		.403**			**.500****	**-.311***	**--**	
**Pb**		-.441*			-.459*		-.473*		**.526****		-.385*			.460*					.443*			--
